# Mechanism of HIV-1 Virion Entrapment by Tetherin

**DOI:** 10.1371/journal.ppat.1003483

**Published:** 2013-07-18

**Authors:** Siddarth Venkatesh, Paul D. Bieniasz

**Affiliations:** Howard Hughes Medical Institute, Laboratory of Retrovirology, Aaron Diamond AIDS Research Center, The Rockefeller University, New York, New York, United States of America; Vanderbilt University School of Medicine, United States of America

## Abstract

Tetherin, an interferon-inducible membrane protein, inhibits the release of nascent enveloped viral particles from the surface of infected cells. However, the mechanisms underlying virion retention have not yet been fully delineated. Here, we employ biochemical assays and engineered tetherin proteins to demonstrate conclusively that virion tethers are composed of the tetherin protein itself, and to elucidate the configuration and topology that tetherin adopts during virion entrapment. We demonstrate that tetherin dimers adopt an “axial” configuration, in which pairs of transmembrane domains or pairs of glycosylphosphatidyl inositol anchors are inserted into assembling virion particles, while the remaining pair of membrane anchors remains embedded in the infected cell membrane. We use quantitative western blotting to determine that a few dozen tetherin dimers are used to tether each virion particle, and that there is ∼3- to 5-fold preference for the insertion of glycosylphosphatidyl inositol anchors rather than transmembrane domains into tethered virions. Cumulatively, these results demonstrate that axially configured tetherin homodimers are directly responsible for trapping virions at the cell surface. We suggest that insertion of glycosylphosphatidyl inositol anchors may be preferred so that effector functions that require exposure of the tetherin N-terminus to the cytoplasm of infected cells are retained.

## Author Summary

The cellular restriction factor, tetherin, prevents HIV-1 and other enveloped virus particles from being disseminated into the extracellular milieu by infiltrating their envelopes and by physically crosslinking them to the cell surface. It is known that tetherin consists of pairs of membrane anchors, situated at either end of a rod-shaped molecule, but how tetherin causes virion tethering has been difficult to unambiguously determine. In this work, we develop genetic and biochemical approaches to probe tetherin molecules that have infiltrated tethered virions. We show that tetherin adopts an “axial” configuration in its functional state, with a pair of membrane anchors situated at one end of the rod-like structure inserted into a tethered virion. While either end of the rod can be inserted into a virion, there is a preference for the insertion of its lipid (glycosylphosphatidyl inositol) modified carboxyl-terminus into virion envelopes. These studies demonstrate unequivocally that the tetherin molecule itself is directly responsible for trapping virions, and dissect the molecular mechanism underpinning its antiviral activity.

## Introduction

Cells have evolved numerous defense measures to inhibit the replication of infectious agents. In animal cells, sensing of viruses by pattern recognition receptors leads to interferon production and signaling, which induces the expression of hundreds of interferon-stimulated genes (ISGs) in infected and bystander cells [1]–[3]. Among these are several classes of autonomously acting proteins (the APOBEC3 proteins, TRIM5 proteins, tetherin and SAMHD1). These proteins are popularly termed “restriction factors”, and are considered to comprise an intrinsic immune system [4] or a specialized arm of conventional innate immunity. Recent efforts have revealed that these proteins directly inhibit the replication of viruses via remarkably divergent and elegant mechanisms of action [5], [6].

Tetherin (also known as BST-2, CD317, or HM1.24) is a type II membrane glycoprotein whose expression is strongly upregulated by type I interferon in most cell types. Tetherin expression causes the physical entrapment of nascent mature enveloped virions at the cell surface [7]–[11]. Structurally, tetherin comprises of a short N-terminal cytosolic tail, a single pass transmembrane helix, an extracellular domain that is predominantly alpha helical [12]–[15], and has three extracellular cysteine residues stabilizing parallel homodimer formation via disulphide bridges. Tetherin is also modified at its C-terminus by a glycosylphosphatidylinositol (GPI) membrane anchor [16], [17].

A few pieces of evidence suggest that tetherin acts directly and autonomously to trap virions at the cell surface. First, trapped virions can be liberated from the cell surface by treatment with the protease subtilisin A, indicating that protein is an essential component of the tethers [18]. In such experiments, tetherin fragments can be found in subtilisin-liberated virions [19]. Second, inactive tetherin proteins in which one of the two membrane anchors is removed are efficiently incorporated into virions [19]. Third, fluorescent and electron microscopic analyses demonstrate that tetherin is localized at sites of virion entrapment [19]–[22]. Fourth, an artificial tetherin protein assembled from heterologous protein domains that have similar configuration but no primary sequence homology to tetherin, recapitulates tetherin function [19]. Taken together these findings suggest that (i) the biological activity of tetherin can be ascribed to its overall configuration rather than its primary sequence and (ii) tetherin does not require specific cofactors or the recognition of specific viral components to cause virion entrapment. These findings are difficult to reconcile with complex models in which tetherin might act as a virion sensor to induce other factors that have tethering activity. Rather, they are more easily explained by the idea that tetherin acts autonomously and directly to trap virions, simply as a consequence of being incorporated into the lipid envelope of virions as they bud through cell membranes. Consistent with these arguments, tetherin exhibits antiviral activity against a broad spectrum of enveloped virions whose proteins have essentially no sequence homology [23]–[30].

Another argument in favor of the notion that tetherin acts rather nonspecifically to trap enveloped virions arises from the mechanisms that viruses have evolved to evade tetherin action. Rather than acquiring viral protein sequence changes that might enable escape from interaction with tetherin, viral proteins have instead adapted to gain interaction with, and thereby antagonize, tetherin. For example, the HIV-1 accessory protein Vpu interacts with the tetherin transmembrane domain [31]–[36], and employs surface downregulation [37]–[41] and degradation [32], [42]–[45] to antagonize tetherin. Additionally, the SIV Nef proteins [46]–[49], the KSHV K5 protein [27], [50], and the HIV-2 Env [38], [51], SIV_MAC_ Env [52] and Ebola Env [53], [54] proteins have adapted to counteract tetherin proteins in their hosts by targeting different portions of the tetherin cytoplasmic tail or ectodomain.

**Figure 1:**
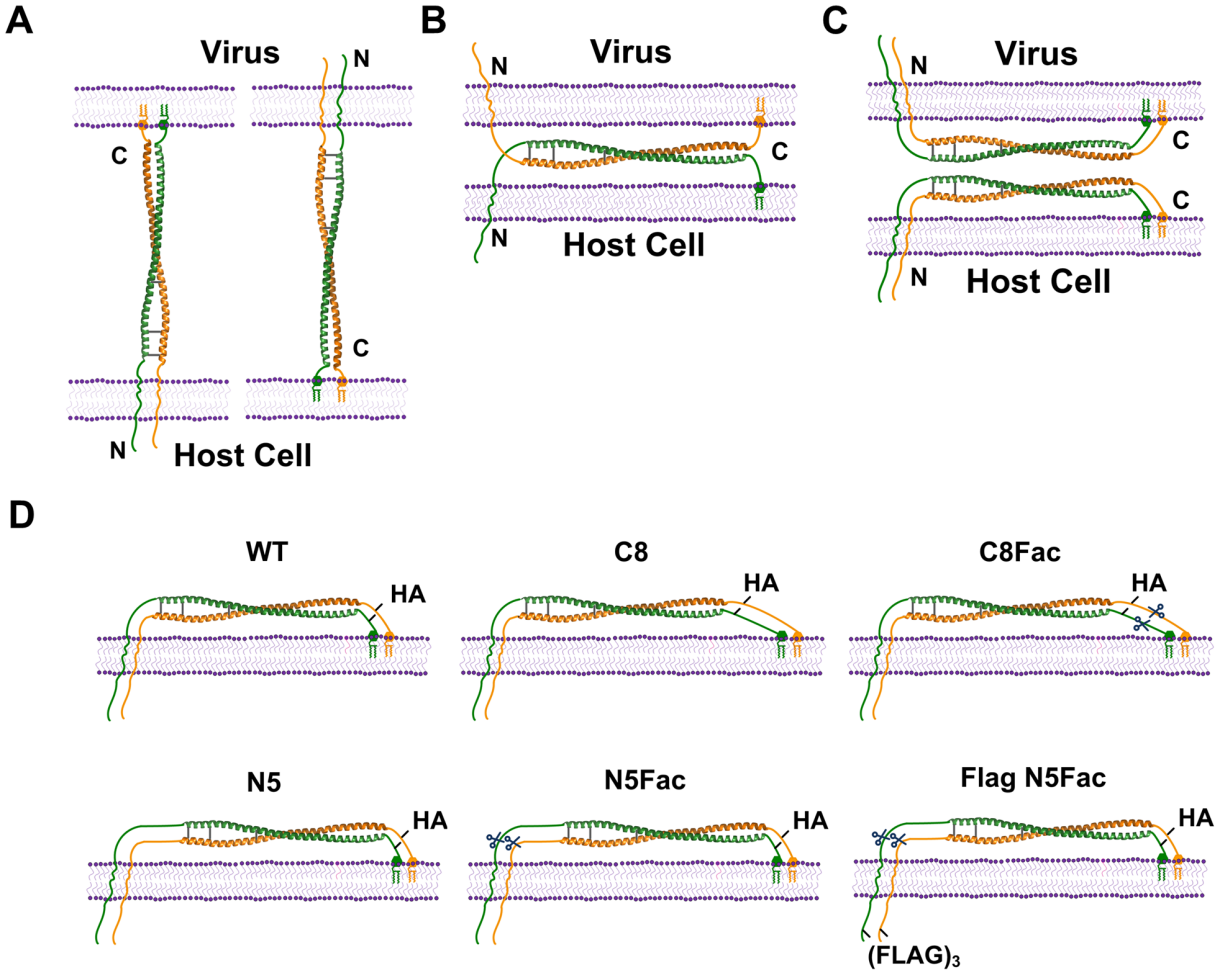
Models illustrating the possible configurations adopted by tetherin during virion entrapment, and the modified tetherin proteins designed to investigate tetherin configuration. (**A**) Tetherin dimers might trap virions at the cell surface via the infiltration of one pair of membrane anchors into the viral envelope, while the other pair remains rooted in the infected cell membrane (axial configuration). ‘N’ and ‘C’ represent the N- and C- termini of tetherin, respectively. (**B**) Tethering might also be achieved through the separate partitioning of dimerized tetherin molecules into virion and cell membranes (equatorial configuration). (**C**) Tethering might be mediated by the non-covalent association of tetherin dimers that have both pairs of anchors embedded in virion envelopes or cell membranes. (**D**) Schematic representation of the panel of modified tetherins proteins designed to deduce the configuration of tetherin during HIV-1 restriction. The C8 and N5 proteins have linker sequences inserted at the C- and N-terminus of the tetherin extracellular domain, respectively. The Factor Xa cleavage site is indicated by scissors, and the cysteines that stabilize homodimerization are indicated as lines. Diagrams were constructed using PDB entry 3MQC to represent the tetherin extracellular domain.

One question that remains incompletely addressed is the precise molecular mechanisms by which tetherin exerts its antiviral activity. As discussed above, a preponderance of the evidence support a direct tethering mechanism, wherein tetherin dimers infiltrate the lipid envelope of assembling particles [19]–[21]. However, while previous biochemical analyses [19] and structural studies [12]–[15] indicate that tetherin forms a rod-like structure with membrane anchors at either end, the configuration adopted by the tetherin protein during entrapment is unknown. For example, because the membrane anchors are spatially separated from each other, it is possible that one pair of anchors partitions into the lipid envelope of assembling particles, while the other pair remains rooted in the plasma membrane of the infected cell (axial configuration, Figure 1A). In this configuration, each tetherin dimer could potentially link viral and cell membranes in either “polarity”, i.e. with N-termini inserted into either the infected cell or the assembling particle. Other obvious possibilities by which entrapment might be achieved would be via the separate partitioning of dimerized tetherin molecules into virion and cell membranes (equatorial configuration, Figure 1B) or the non-covalent oligomerization of tetherin dimers that have both pairs of anchors embedded in either virion envelopes or cell membranes (Figure 1C).

Because a protected, β-mercaptoethanol-sensitive, dimeric amino-terminal tetherin fragment can be recovered from virions that have been liberated by protease treatment, it appears that at least some trapped virions are infiltrated by both N-termini of a parallel tetherin homodimer, favoring the models shown in Figure 1A or 1C [19]. Moreover, a tetherin variant that lacks a GPI anchor preferentially localizes to sites of viral budding, suggesting that the tetherin N-terminus provides the dominant driving force for infiltration into budding virions [19]. These results have also been supported by other studies involving super-resolution microscopy [22]. Nevertheless, it remains a challenge to establish if any of the aforementioned configurations are adopted during the retention of virions, or whether the contribution of any one outweighs that of the others.

Herein, we have employed quantitative biochemical experiments and engineered tetherin proteins to demonstrate conclusively that tetherin acts directly to trap virions and to elucidate the mechanisms of virion entrapment. Specifically, we placed epitope tags and cleavage sites for the site-specific protease Factor Xa at strategic positions in the tetherin molecule. Virions that were tethered at the cell surface by these modified tetherin proteins were liberated upon specific protease treatment and analyzed. Our results demonstrate that tetherin dimers trap virions by adopting the axial configuration (Figure 1A), with either transmembrane domains or GPI anchors capable of infiltration into assembling particles. Quantitative analyses suggested that, on an average, a few dozen tetherin dimers are involved in trapping each virion and that there is a ∼3–5 fold preference for a tetherin orientation in which the GPI anchored C-terminus rather than the transmembrane domain is inserted into a tethered particle. Taken together, our biochemical experiments constitute the most compelling evidence to date that tetherin is directly responsible for trapping virions at the cell surface and that this is achieved using axially positioned tetherin homodimers, that are primarily configured with their GPI anchored C-termini inserted into virions.

## Results

### Antiviral activity of modified tetherin proteins

In this study, we endeavored to develop biochemical assays to unequivocally determine whether tetherin acts as a direct tether in trapping virions, and to determine the configuration of tetherin dimers that are engaged in virion entrapment. A variety of approaches, including hydropathy analyses, fusion with reporter enzymes, or the insertion of target sites for proteases, antibodies and chemical modifiers, have been used to deduce membrane protein topology [55]. For example, the insertion of Factor Xa cleavage sites into hydrophilic loops has proven to be useful in such analyses [56], [57]. We adapted these approaches by engineering modified human tetherin proteins that carried (i) single cleavage sites for Factor Xa and (ii) epitopes such as hemagglutinin (HA) and FLAG tags positioned either N- or C-terminal to the Factor Xa site (Figure 1D). Previous experiences with modified tetherin proteins led to the expectation that these alterations should have no or modest effects on antiviral activity [7], [19].

Initial experiments in which Factor Xa sites alone were incorporated into Tetherin resulted in proteins that were somewhat refractory to proteolysis (unpublished observations). Hence, we reasoned that the introduction of flexible linkers into its primary sequence might facilitate access to the cleavage site, and increase the efficiency of proteolysis. Therefore, we generated a panel of proteins in which we inserted five and eight GGGGS peptide linker units into the extracellular domain of tetherin, either N-terminal (at amino acid 50) or C-terminal (at amino acid 157) to the predicted coiled-coil domain. The GGGGS peptide is predicted to be unstructured because the glycine residues impart flexibility, and the polar serine residue permits hydrogen bonding to the solvent [58], [59].

**Figure 2:**
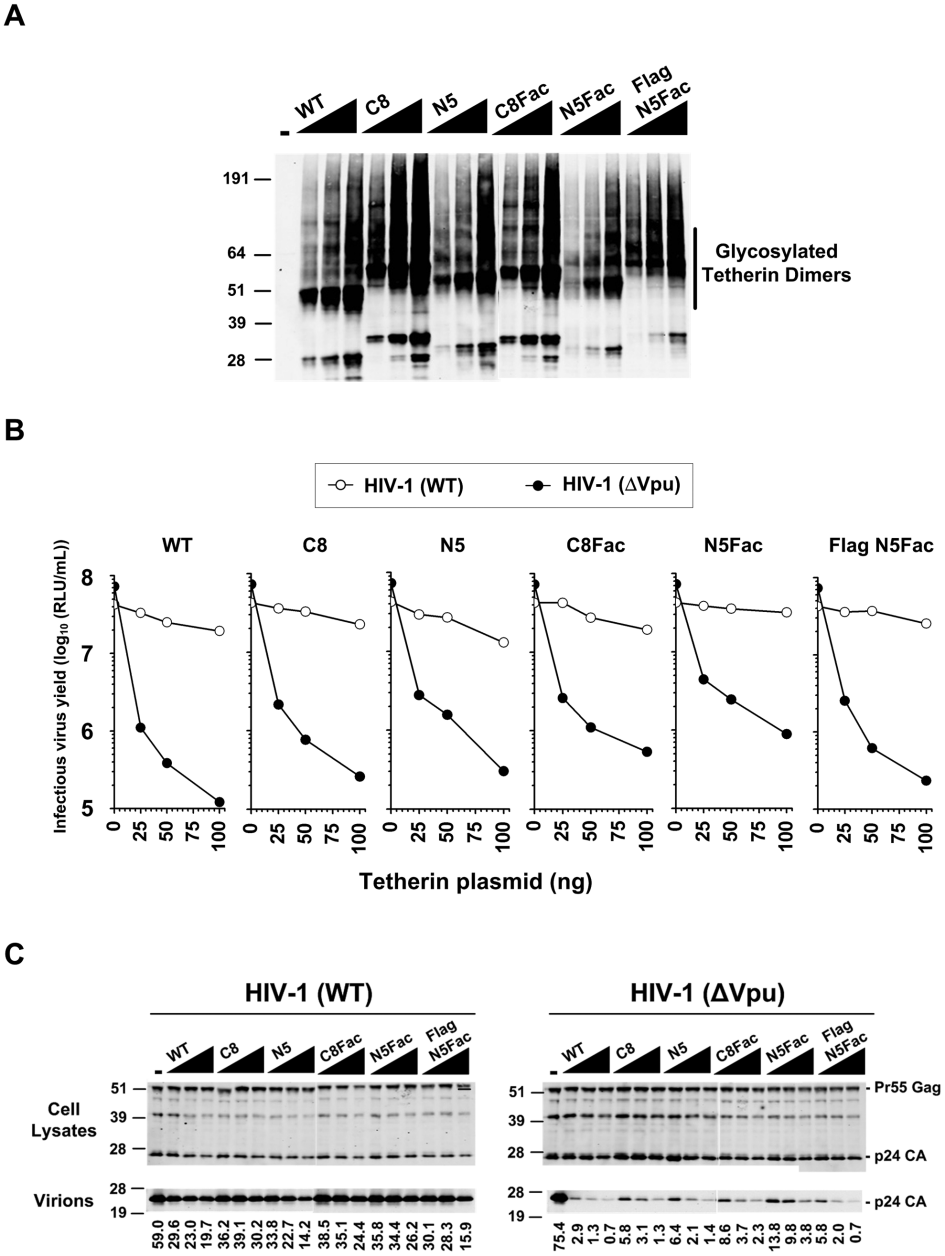
Antiviral activity of the panel of modified tetherin proteins. (**A**) Western blot analyses of the 293T cell lysates that were cotransfected with a Vpu-deficient (HIV-1 ΔVpu) proviral pNL4-3 plasmid along with varying amounts of the indicated modified tetherin proteins. All samples were probed with an anti-HA antibody. (**B**) 293T cells were cotransfected with WT (HIV-1 WT) or Vpu-deficient (HIV-1 ΔVpu) proviral pNL4-3 plasmids along with varying amounts of the indicated modified tetherin proteins. Infectious virion yield was measured by inoculating HeLa-TZM indicator cells with culture supernatant and is given as the logarithm to the base 10 of the relative light units (RLU). (**C**) Western blot analyses of transfected 293T cell lysates and virions corresponding to the above panel. All samples were probed with an anti-CA antibody. The numbers at the bottom represent measurement of CA protein levels in virion pellets (LI-COR).

Among the panel of linker modified tetherin proteins, we determined that the proteins with eight linker units C-terminal to the coiled-coil (C8, Figure 1D) and five linker units N-terminal to the coiled-coil (N5, Figure 1D) were expressed at comparable levels to WT tetherin (Figure 2A). Note that Tetherin is heterogeneously glycosylated, and because the cells were lysed in non-reducing buffer, the tetherin proteins migrated primarily as a smear of dimeric species [19] (Figure 2A). To examine the antiviral activity of the linker-modified tetherin proteins, we co-expressed an HIV-1 proviral plasmid (HIV-1(WT)) or its Vpu-deficient counterpart (HIV-1(ΔVpu)) along with varying amounts of plasmids expressing WT tetherin or one of the modified tetherin proteins. Hereafter, WT tetherin (Figure 1D) refers to a previously described construct that harbors an HA epitope tag at amino acid 155 in the extracellular domain, but retains the antiviral activity of the untagged, endogenous protein [7]. As expected, WT tetherin potently inhibited the release of HIV-1 (ΔVpu) in a dose-dependent manner, while only marginally affecting the release of HIV-1 (WT) (Figure 2B, C). Importantly, the C8 and N5 tetherin proteins were only modestly impaired in their antiviral activity compared to WT tetherin as determined by infectious virion yield and extracellular particulate CA protein measurements (Figure 2B, C) and the levels of cell-associated Gag protein were unaffected by the expression of the tetherin proteins (Figure 2C). Thus, the insertion of linker sequences into tetherin was well tolerated with little effect on antiviral activity.

We next programmed the C8 and N5 tetherin proteins with single Factor Xa cleavage sites. The rationale was that these proteins (referred to hereafter as C8Fac and N5Fac respectively, Figure 1D) would differ in the relative ordering of the HA epitope tag and the protease site. Thus, the epitope tag is positioned N-terminal to the protease site in the C8Fac protein, whereas it is positioned C-terminal to the protease site in the N5Fac protein. In addition to the C8Fac and N5Fac proteins that carried only one epitope tag, we also appended the N-terminus of the N5Fac construct with three tandem FLAG epitope tags. This manipulation results in FLAG and HA epitope tags flanking the protease site (Flag N5Fac, Figure 1D). The use of three FLAG tags in tandem reportedly enhances signal intensity by ∼10–20-fold [60].

Analysis of the antiviral activity of the Factor Xa site-modified tetherin proteins revealed that the C8Fac and N5Fac proteins were only slightly impaired in activity relative to WT tetherin, while the Flag N5Fac protein was nearly indistinguishable in antiviral activity to WT tetherin (Figure 2B, C). The C8Fac and N5Fac proteins were expressed at slightly lower levels than the C8 and N5 proteins respectively (Figure 2A), and were proportionately impaired in antiviral activity (Figure 2B, C). Interestingly, despite harboring more tags as compared to any of the other modified tetherin proteins, the Flag N5Fac protein was virtually as potent as WT tetherin, and expressed at levels indistinguishable from WT tetherin. Vpu antagonized all modified tetherin proteins and restored the yield of extracellular virions (Figure 2B, C). Thus, all modified tetherin proteins mimicked the biological activity and Vpu sensitivity of WT tetherin.

**Figure 3:**
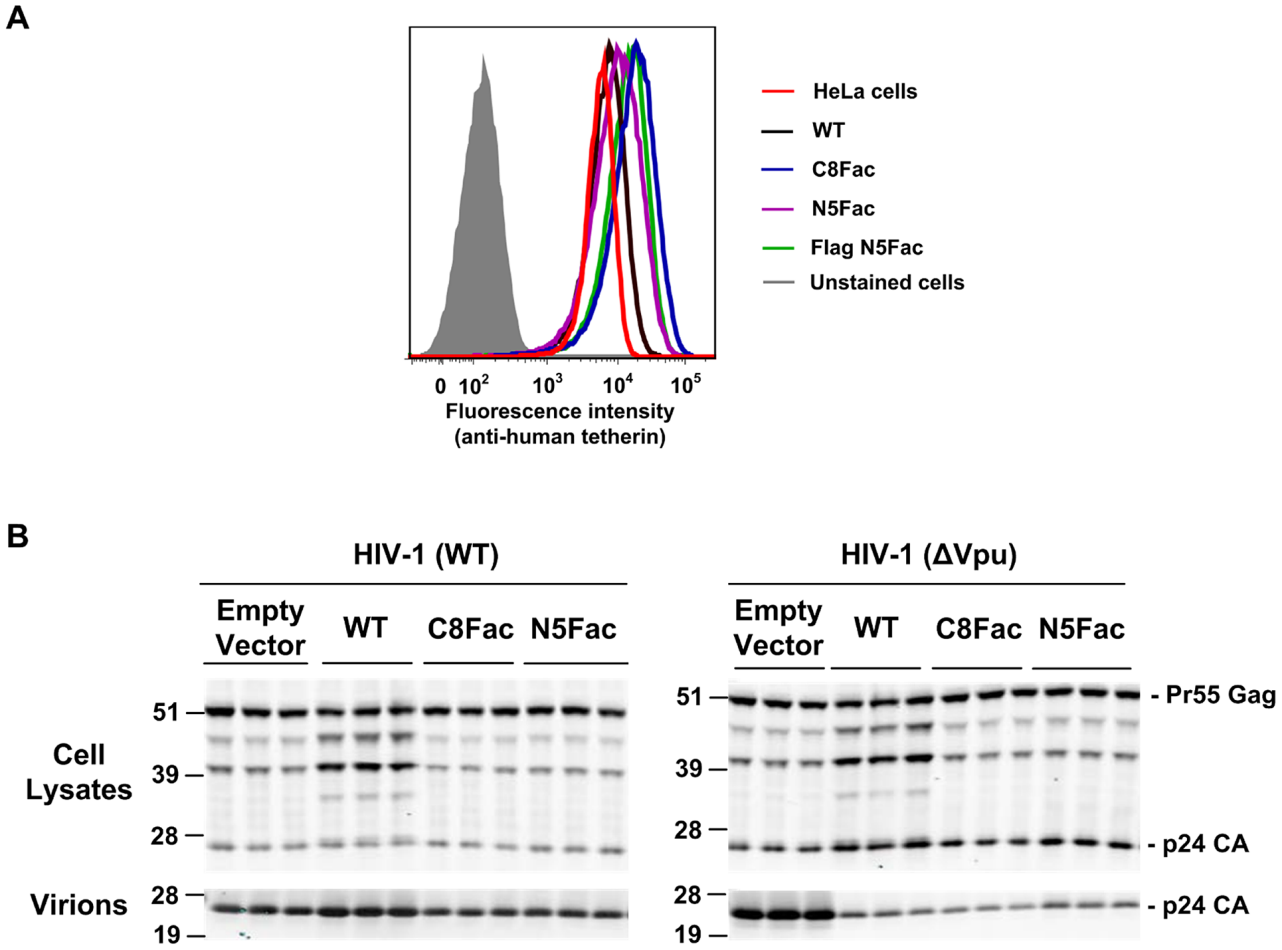
Antiviral activity of modified tetherin proteins in stable cell lines. (**A**) 293T cells stably expressing the modified tetherin proteins were analyzed by flow cytometry to determine the relative surface expression levels of tetherin, using a mouse anti-human tetherin antibody. The mean fluorescence intensity (MFI) for endogenous tetherin in HeLa cells was 5000, while the MFIs for WT, C8Fac, N5Fac and Flag N5Fac tetherin proteins were 6200, 15000, 8800, and 12000 respectively. (**B**) Western blot analyses of the stable 293T cell lysates and virions harvested from them, following infection with HIV-1 or its Vpu-deficient counterpart at a MOI of 1. All samples were probed with an anti-CA antibody. The three lanes for each tetherin protein are replicates of the experiment.

We next generated a panel of 293T cells that stably expressed the epitope-tagged Factor Xa-cleavable tetherin proteins. The levels of cell surface tetherin in these stable cell lines was assessed by flow cytometry using a monoclonal antibody that recognizes the extracellular region of human tetherin. Importantly, the surface expression levels of the WT and modified tetherin proteins were quite similar to each other, varying over a 2.5-fold range (mean fluorescent intensities were 6200, 15000, 8800, and 12000 for WT, C8Fac, N5Fac and Flag N5Fac tetherin proteins, respectively) and were only 1.5 to 3-fold greater than that of the endogenous protein in HeLa cells, a prototype tetherin-positive cell line (mean fluorescent intensity = 5000, Figure 3A). Additionally, we verified that the engineered tetherins exhibited antiviral activity in the stable cell lines using single-cycle HIV-1 replication assays (Figure 3B). As expected, both the WT and the modified tetherin proteins inhibited the release of virions from infected cells, but did not affect cell associated Gag protein expression (Figure 3B). Also, the expression of Vpu reversed the inhibitory effect of the modified tetherin proteins (Figure 3B).

### An assay to probe the configuration and topology of tetherin during HIV-1 particle entrapment

**Figure 4:**
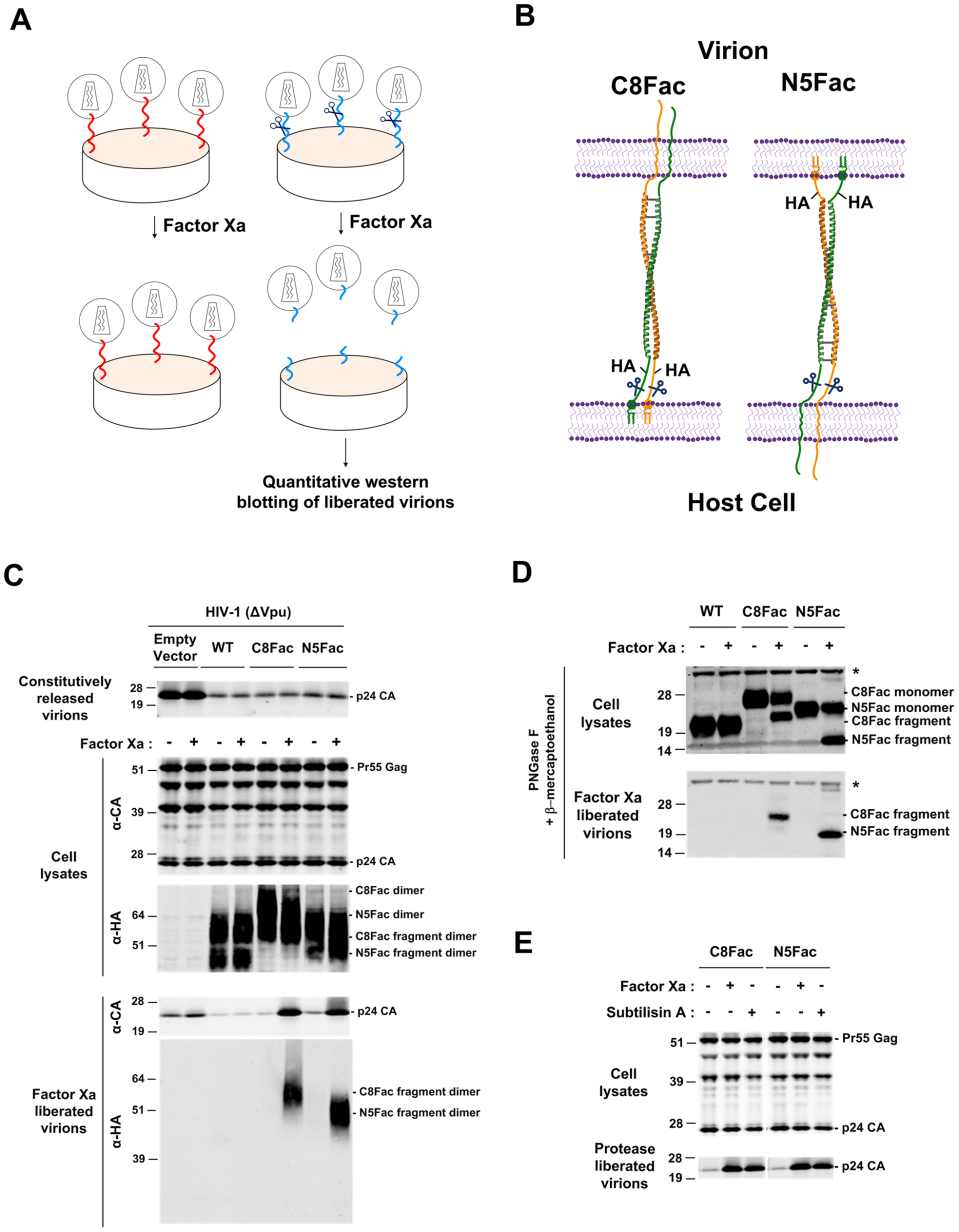
Axially oriented tetherin homodimers directly trap virions. (**A**) Schematic representation of the protease-induced virion release assay. Modified tetherin dimers programmed with a protease cleavage site (scissors) are indicated as blue helices, while the WT tetherin dimers are indicated as red helices. If trapped viruses are liberated upon protease treatment only when tetherin is programmed with a protease site, it confirms a role for tetherin as a direct tether in virion entrapment. The liberated virions are subjected to quantitative western blotting analyses to estimate the numbers of tetherin dimers associated with a single virion and their orientation. (**B**) Schematic representation of the polarities that would be adopted by the C8Fac and N5Fac proteins if HA-tagged proteolytic fragments are observed to partition with liberated virions. (**C**) Western blot analyses of virions, 293T cells stably expressing C8Fac and N5Fac tetherin proteins, and virions liberated upon Factor Xa treatment. The samples were probed using anti-HA and anti-CA antibodies. (**D**) Western blot analyses of PNGase-F-treated cells and liberated virions from the above panel. The samples were probed using an anti-HA antibody. Stars indicate non-specific bands. (**E**) Western blot analyses of 293T cells stably expressing C8Fac and N5Fac tetherin proteins and virions liberated upon Factor Xa or subtilisin A treatment. The samples were probed using an anti-CA antibody.

Our previous studies have employed a protease “stripping” assay [18], [61] in which a relatively nonspecific protease (subtilisin A) was used to demonstrate that tetherin causes virions to become entrapped on cell surfaces by a protein based tether. The logic underpinning the assay described herein was that if the tetherin protein itself functions as the direct tether, then treatment of cell surfaces with a specific protease (Factor Xa) would trigger the release of virions, only when tetherin was programmed with a Factor Xa cleavable site (Figure 4A). Moreover, cleavage should result in partitioning of the epitope-tagged proteolytic fragments either into the liberated virions or the infected cells. Because the epitope tags were strategically positioned relative to the protease site, topological information could be deduced about tetherin in its functional state (Figure 4B). However, because we expect that only a minority of the tetherin molecules on the cell surface would actually be involved in tethering virions, only fragments that are found in virions should be regarded as informative with respect to tetherin topology during virion entrapment.

Note that if tetherin adopts the equatorial configurations depicted in Figure 1B or 1C then we would not expect Factor Xa cleavage to result in virion release, because the cleavage sites are positioned outside the region of tetherin-tetherin interaction, in the rod like portion of the molecule. Indeed, the cleavage sites are positioned in artificially introduced sequences whose insertion did not perturb tetherin function (Figure 2, 3). Conversely, if tetherin adopts the axial configuration in virion tethers, then Factor Xa cleavage should result in virion release. Moreover, if as depicted in Figure 4B, the HA-tagged proteolytic fragments partition with virions that are liberated from Factor Xa-treated, C8Fac-expressing cells, it would suggest that tetherin dimers exist with their N-termini inserted into the interior of the virion. Conversely, if HA-tagged proteolytic fragments partition with virions that are liberated from the N5Fac cell line, we would deduce that tetherin dimers exist with their GPI anchors embedded in the virion membrane. If, however, tetherin dimers adopt both polarities, then HA-tagged proteolytic fragments would be observed in virions liberated by Factor Xa from both C8Fac and N5Fac expressing cell lines.

We first investigated the utility of this approach using cell lines expressing the single epitope tagged C8Fac and N5Fac tetherin proteins. Cells were infected with single-cycle, Vpu-deficient HIV-1, and constitutively released particles were harvested from culture supernatants. Thereafter, the monolayer of cells was treated with Factor Xa, and then the cell lysates and any liberated virions were also harvested. As before, infected tetherin-negative control cells constitutively released comparatively high levels of virions into the culture supernatant, while virion yield from cells expressing WT, C8Fac or N5Fac tetherin proteins was substantially reduced (Figure 4C). The levels of HIV-1 Gag expression in cell lysates were uniform (Figure 4C).

Incubation in Factor Xa cleavage buffer alone resulted in the release of only low levels of pelletable CA from tetherin-deficient cells. This may have represented virion particles that were constitutively released during incubation, or virions that were loosely adhered to the cell surface (Figure 4C). Even lower levels of particles were released from cells expressing the WT, C8Fac or N5Fac tetherin proteins that were incubated in Factor Xa cleavage buffer alone. Strikingly however, Factor Xa treatment of the C8Fac and N5Fac resulted in the release of substantial amounts of particulate CA (Figure 4C). Crucially, Factor Xa treatment of tetherin-negative or WT tetherin expressing cells did not increase particle release over the low background levels that were observed in the absence of protease, underscoring the strict requirement for a Factor Xa-cleavable tetherin in Factor Xa-induced virion release (Figure 4C). Notably, proteolytic fragments of tetherin were observed in virions released by Factor Xa from both C8Fac and N5Fac expressing cells and these virion-associated fragments were consistent with the incorporation of tetherin dimers therein. These dimers were the only tetherin species that were detectable on non-reducing SDS PAGE gels (Figure 4C).

Because tetherin is intrinsically heterogeneous, due to variable glycosylation as well as dimer formation, it was difficult to assess the extent of Factor Xa cleavage in cell lysates (Figure 4C, center panel), or to unambiguously demonstrate that only cleaved tetherin fragments were present in Factor Xa liberated virions (Figure 4C, bottom panel). Therefore we treated cell and virion lysates with PNGase-F and repeated the western blot analyses under reducing conditions. We observed that the HA-tagged proteolytic fragments (predicted molecular weights of ∼20.8 kDa and ∼17 kDa for C8Fac and N5Fac respectively) could be resolved from the full-length molecules (∼24.7 kDa and ∼23.8 kDa for C8Fac and N5Fac respectively) (Figure 4D). This analysis revealed that about half of the cell-associated C8Fac and N5Fac protein was cleaved by Factor Xa that was applied to the cell surface. The incomplete cleavage may have been due to the intracellular localization of a fraction of the tetherin protein. As expected, no proteolysis of the WT tetherin protein was observed (Figure 4D). Notably, only the cleaved tetherin protein was found in PNGase-F-digested virion lysates, consistent with the notion that tetherin cleavage by Factor Xa was necessary for virion release in this assay. To assess the efficiency of tetherin cleavage and virion release by Factor Xa, we compared the levels of virion released from C8Fac and N5Fac expressing cell lines following treatment with Factor Xa or with subtilisin A (Figure 4E). Similar amounts of virions were released by the site-specific and non-specific proteases. This result suggested that tetherin cleavage and virion release caused by Factor Xa was quite efficient. It also suggested that it was unlikely that a significant fraction of virions are retained using alternative configurations of tetherin (Figure 1) in which virion release might be resistant to Factor Xa treatment.

Overall, these results strongly suggested that tetherin traps virions by adopting the axial configurations depicted in Figure 1A. Moreover, because HA-tagged proteolytic fragments from both C8Fac and N5Fac tetherin proteins partitioned with virions these data suggested that both polarities depicted in Figure 1A are adopted by tetherin during virion entrapment.

### Estimates of the number of tetherin dimers associated with tethered virions

**Figure 5:**
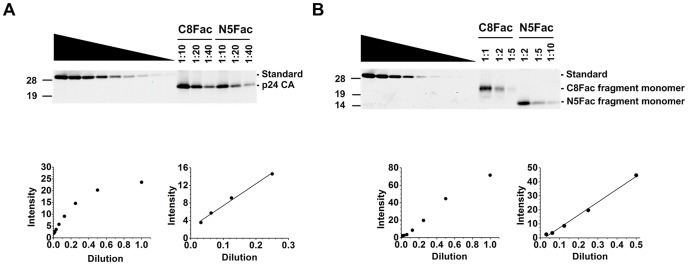
Quantitative Western blot analyses of Factor Xa-liberated virions. (**A**) Western blot analyses of serially diluted lysates of 293T cell expressing the CA-HA-Flag protein along with the PNGase-F-treated, Factor Xa-liberated virion lysates (top panel). The samples were probed with an anti-CA antibody. The CA band intensities for the control protein were analyzed using a LiCOR Odyssey scanner and were plotted against the dilution (lower left panel). Regression analysis was performed over the linear range of signal intensities (lower right panel) and the unknown amounts of CA in the PNGase-F-treated liberated virion lysates were deduced by interpolation of the standard curve. (**B**) Same as (**A**), except that the samples were probed with an anti-HA antibody. The star indicates a non-specific band.

To estimate the number of tetherin dimers that were involved in the entrapment of a single virion, we used a quantitative western blotting approach and PNGase-F-digested virion lysates to measure the relative number of CA and HA epitopes associated with virions that had been tethered by the C8Fac and N5Fac proteins, and then released by Factor Xa cleavage. First, we generated an appropriate internal standard protein to enable relative quantitation. This standard consisted of a fusion protein that comprised the HIV-1 p24CA protein, appended at its C-terminus with three tandem FLAG tags and an HA epitope tag. Thus, this single protein included each of the epitopes that we planned to probe, at a stoichiometric ratio of 1∶1∶1 and could be used as a standard to compare the relative numbers of HA and CA epitopes in tethered virions liberated from C8Fac and N5Fac expressing cells. Specifically, serial dilutions of cell lysates expressing the HA-Flag-CA protein were run on SDS-PAGE gels, blotted onto membranes and probed with antibodies against CA and HA. The band intensities were analyzed using a LiCOR Odyssey scanner (Figures 5A, B), and regression analysis was performed over the linear range of signal intensities (Figures 5A, B). Dilutions of the PNGase-F-treated virion lysates recovered from C8Fac and N5Fac expressing cells that also yielded band intensities in the linear range of the assay were resolved on the same gel as the standard, and the relative amounts of CA and HA epitope in each samples were deduced by interpolation using the standard curves (Figures 5A, B).

HIV-1 virions have been reported to contain between 1000–5000 copies of the Gag protein, of which only a fraction contribute to core formation [62]–[66]. We calculated our estimates of tetherin dimers per virion based on the extremities of this range (Table 1). Thus, if each virion contains 1000 CA protein molecules, we estimate that 16±5 dimers of the N-terminus of C8Fac and 71±26 dimers of the C-terminus of N5Fac tetherin dimers were associated with a single tethered virion (Table 1). Conversely, if a single virion contains 5000 CA epitopes, then we estimate that 80±25 dimers of the N-terminus of C8Fac and 355±130 dimers of the C-terminus of N5Fac tetherin dimers were associated with a single tethered virion. Thus these numbers suggested a preference (∼4 to 5-fold) for the insertion of the GPI-anchored tetherin C-terminus, rather than the N-terminal transmembrane domain into virions. Note that the larger number of HA tags associated with virions in the case of N5Fac cannot be explained by differences in tetherin expression levels. In fact, there were lower levels of N5Fac on cell surfaces (MFI = 8800, Figure 3A) as compared to the C8Fac protein (MFI = 15000, Figure 3A).

**Table 1:** Quantitative western blotting analysis of virions tethered by the C8Fac and N5Fac proteins.

	Tetherin dimers per virion		Tetherin dimers per virion	
	(assuming 1000 CA molecules/virion)		(assuming 5000 CA molecules/virion)	
	C8Fac	N5Fac	C8Fac	N5Fac
Experiment 1	16	42	80	210
Experiment 2	21	80	105	400
Experiment 3	11	92	55	460
Mean ± Standard Deviation	16±5	71±26	80±25	355±130

### Preferential insertion of tetherin C-termini into HIV-1 particles during virion entrapment

**Figure 6:**
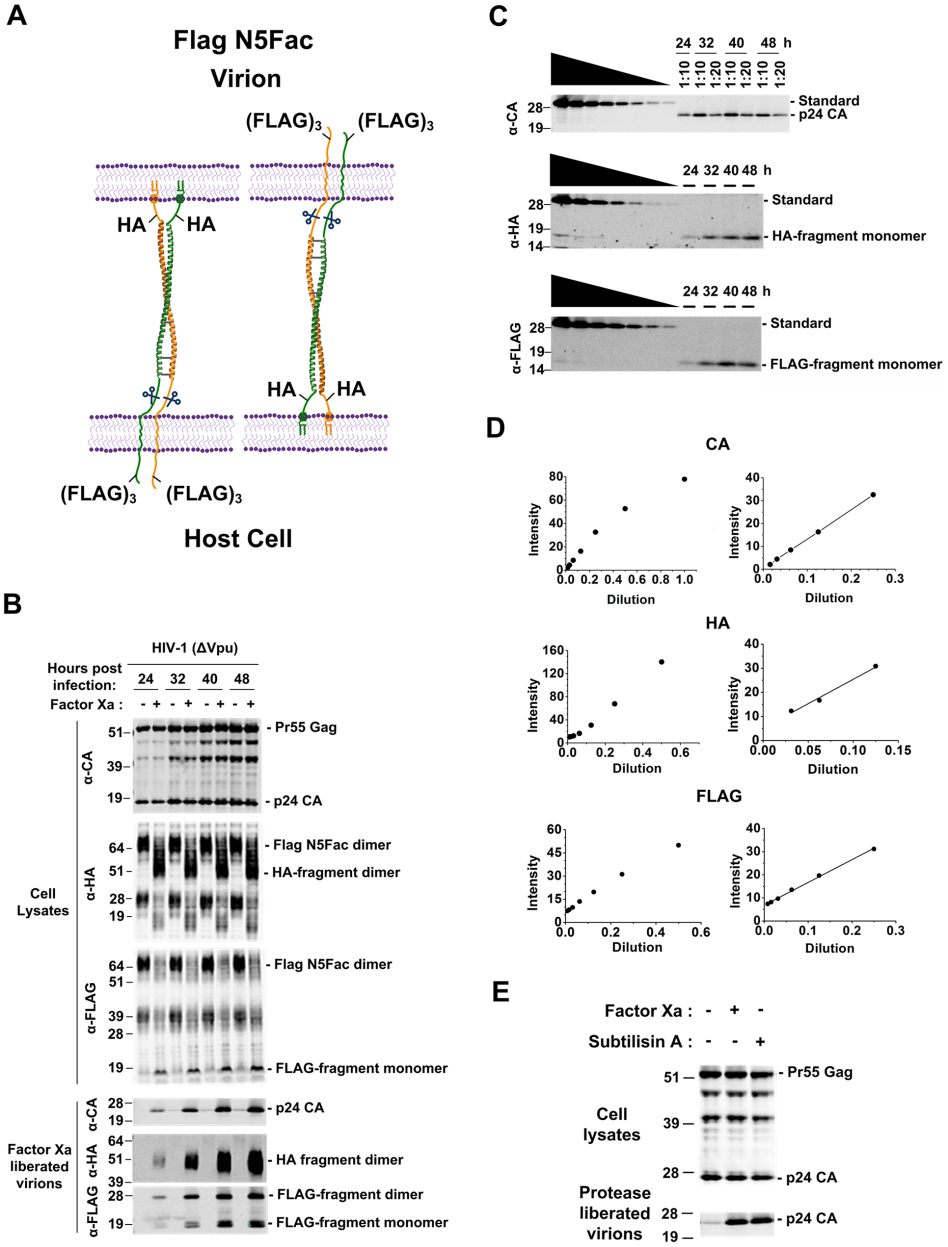
Preferential insertion of tetherin C-termini into virion envelopes. (**A**) Schematic representation of the polarities that would be adopted by the Flag N5Fac protein if HA- or FLAG-tagged proteolytic fragments are observed to partition with liberated virions. (**B**) Western blot analyses of 293T cells and liberated virions, obtained by the protease treatment of Flag N5Fac cells at various time points following infection. The samples were probed using anti-HA, anti-FLAG and anti-CA antibodies. (**C**) Western blot analyses of 293T cells expressing serial dilutions of the CA-HA-Flag protein and the PNGase-F-treated liberated virion lysates. The samples were probed with anti-CA, anti-HA and anti-FLAG antibodies. (**D**) The CA, HA and FLAG band intensities for the control protein were determined using a LiCOR Odyssey scanner and were plotted against the dilution (left panels). Regression analysis was performed over the linear range of signal intensities (right panels), and the unknown amounts of CA, HA and FLAG epitopes in the PNGase-F-treated, Factor Xa-liberated virion lysates were deduced by interpolation from the standard curves. (**E**) Western blot analyses of 293T cells stably expressing the Flag N5Fac tetherin protein and virions liberated upon Factor Xa or subtilisin A treatment. The samples were probed using an anti-CA antibody.

The aforementioned experiments indicated that tetherin directly tethers HIV-1 particles in an axial configuration (Figure 1A) and suggested that both polarities, with either N- or C- termini inserted into virions contribute to antiviral activity. However, it was possible that the two different estimates for the numbers of tetherin molecules inserted into virions with each polarity might reflect intrinsic differences in the properties of the two different tetherin molecules used (C8Fac and N5Fac). Therefore, we quantitated tetherin insertion into virions in a second set of experiments employing a single tetherin species with two different epitope tags on either side of the Factor Xa cleavage site (Flag N5Fac, Figure 1D, Figure 6A). Additionally, we have previously found that virions that accumulate on the surface of cells as a result of tetherin action can sometimes be tethered to each other as well as to the cell surface. This scenario could be the result of virion assembly at sites on the cell surface already occupied by trapped virions and would result in both ends of a tetherin molecule being associated with virions. These events would tend to reduce any indication that tetherin N-or C-termini are preferentially inserted into virion envelopes. Because the accumulation of virions should exacerbate this effect over time, we treated the surface of cells expressing Flag N5Fac with Factor Xa at predetermined time intervals following infection with HIV-1ΔVpu, and quantified HA- and FLAG-tagged proteolytic fragments in liberated virions.

The HIV-1 Gag protein became detectable in infected Flag N5Fac-expressing cell lysates at ∼24 h after infection and levels progressively increased with time thereafter (Figure 6B). Treatment of these infected cells with Factor Xa resulted in a time dependent increase in the amount of recovered virions (Figure 6B). The Factor Xa site is positioned N-terminal to the sites of N-linked glycosylation as well as to the extracellular cysteines in the Flag N5Fac molecule (Figure 1D, Figure 6A) and so the Factor Xa cleavage of the 65–70 kDa dimeric, glycosylated Flag-N5Fac protein yields a cell associated dimeric, glycosylated ∼50–55 kDa αHA reactive species as well as a cell associated monomeric, nonglycosylated 10 kDa α-FLAG reactive species (Figure 6B). Notably, both the dimeric glycosylated ∼50–55 kDa αHA reactive species and the 10kDa α-FLAG reactive species were observed in virions, and their levels in the virion fraction increased with time, approximately in parallel with the increasing yield of Factor Xa liberated virions (Figure 6B). Notably, the N-terminal FLAG tagged fragment of Flag N5Fac was also found in virions in a form that was consistent with the formation of dimers. We hypothesize that this is because the tetherin cytoplasmic tail contains two cysteines that can form disulphide bonds in the interior of virions. Consistent with this idea, only the smaller of the two Flag tagged species was observed when virion lysates were subjected to SDS PAGE gel electrophoresis under reducing conditions (Figure 6C). Additionally, the dimeric glycosylated ∼50–55 kDa αHA reactive species collapsed to a single ∼17 kDa band when samples were deglycosylated with PNGase and reduced (Figure 6C).

We used quantitative western blot analyses of PNGase-F-digested virion lysates to estimate the number of copies of HA- and FLAG-tagged proteolytic fragments per trapped virion (Figure 6C, D). Again we used the FLAG-HA-CA protein as a standard to determine the relative numbers of HA, FLAG and CA epitopes in the virions liberated from Flag N5Fac expressing cells. Although tethered virions could be recovered from the surface of Flag N5Fac expressing cells beginning at 24 h after infection, we could not make reliable estimates of the HA and FLAG fragments at this time point, as they were present at levels that were close to the limit of detection. However, we could make reasonably robust estimates of the levels of incorporation of HA- and FLAG-tagged fragments into virions beginning at 32 h after infection.

Importantly, the number of FLAG-tagged dimers that were estimated to be present in virions (assuming 1000 CA molecules per virion) tethered by Flag N5Fac (11±3 [at 32 h] to 16±6 [at 48 h], Figure 6C, D, Table 2) correlated quite well with the number of HA-tagged dimers present in virions tethered by C8Fac (16±5 [at 48 h], Table 1). Similarly, the number of HA-tagged dimers in tethered virions recovered from the Flag N5Fac expressing cells (34±18 [at 32 h] to 55±28 [at 48 h]) (Figure 5D, Table 2) correlated quite well with the number of copies of HA-tagged dimers in tethered virions recovered from the N5Fac expressing cells (71±26 at 48 h) (Table 1). Notably, we estimated that the virions liberated from Flag N5Fac expressing cells carried ∼3 to 4-fold more HA tags than FLAG tags, again suggesting that axially configured tetherin dimers infiltrate assembling particles, with a tendency to embed their C-termini rather than their N-termini in tethered virions. Also noticeable was a marginal trend for the appearance of increasing numbers of tetherin molecules per virion over time. This trend was not statistically significant and could be due to some unknown bias in the measurements. However, it is also possible that virions with smaller numbers of tetherin molecules are more readily released, leading to the preferential accumulation of virions with greater numbers of tetherin molecules on the surface of cells. Finally, to confirm that alternative Factor Xa-resistant configurations of Flag N5Fac tetherin were not responsible for retaining a significant fraction of virion particles, we compared the levels of virions released from Flag N5Fac expressing cells by Factor Xa or by subtilisin A treatment. Similar amount of particles were released by the two proteases, suggesting that axially configured Flag N5Fac tetherin molecules were the major form responsible for virion retention (Figure 6E).

**Table 2:** Quantitative western blotting analysis of virions tethered by the Flag N5Fac protein.

	Tetherin dimers per virion						Tetherin dimers per virion					
	(assuming 1000 CA molecules/virion)						(assuming 5000 CA molecules/virion)					
	HA tags			FLAG tags			HA tags			FLAG tags		
Hours post infection	32	40	48	32	40	48	32	40	48	32	40	48
Experiment 1	47	39	66	14	18	22	235	195	330	70	90	110
Experiment 2	42	43	76	10	11	13	210	215	380	50	55	65
Experiment 3	14	18	23	9	10	12	70	90	115	45	50	60
Mean ± Standard Deviation	34±18	34±14	55±28	11±3	13±4	16±6	170±90	170±70	275±140	55±15	65±20	80±30

## Discussion

We devised a biochemical approach to probe tetherin molecules that have infiltrated virions at the cell surface, with the goal of elucidating the configuration adopted by tetherin during virion entrapment. This approach was based on two previous findings. First, a non-specific protease, subtilisin, could be used to liberate tethered particles from the infected cell's surface [18], [61]. Second, the primary sequence of tetherin can be drastically altered while retaining biological activity [19]. Thus, we employed the site-specific protease Factor Xa to liberate virions trapped by tetherin molecules that were engineered to include its cleavage site. This manipulation gave the approach tight specificity and enabled the unequivocal demonstration that the tetherin protein itself is an essential component of virion tethers. Moreover, the use of a site specific protease to release tethered virions from cell surfaces enabled the preservation of epitope tags inserted into the tetherin ectodomain, allowing us to infer the organization of tetherin molecules in virion tethers. We could use a double epitope-tagged version of tetherin, as well as single epitope-tagged versions to analyze the incorporation of both N- and C-terminal proteolytic fragments into virions, and thereby determine tetherin configuration. Additionally, we constructed a protein standard and performed quantitative western blotting to estimate the numbers of tetherin dimers in each orientation that are associated with trapped virions.

Because virions were efficiently liberated by Factor Xa treatment of N5Fac or C8Fac expressing cells, our data effectively exclude the “equatorial” configuration shown in Figure 1B, as cleavage of the tetherin peptide backbone in this context would leave intact the majority of the bonds holding the virion on the cell surface. Moreover, the fact that tetherin fragments found in virions liberated by Factor Xa were exclusively disulphide linked homodimers also constitutes strong evidence disfavoring this model. While our data do not completely discount the possibility that tetherin multimers adopt the equatorial configuration, with virions becoming trapped via hypothetical noncovalent dimer-dimer interactions (Figure 1C) this scenario appears unlikely for two reasons. First, such a configuration would not be expected to result in virion release upon Factor Xa cleavage, because dimer-dimer interactions would not be expected to be perturbed, particularly since the Factor Xa cleavage site is placed within a foreign spacer sequence whose insertion does not itself perturb tetherin function. Second, the scenario envisaged in Figure 1C would result in precisely equal numbers of tetherin N- and C termini being placed in tethered virions. We found that there were modestly, but clearly, more tetherin C-termini than N-termini in virions, arguing that tetherin N- and C-termini partition separately into virion and cell membranes. Overall our experiments indicated that tetherin homodimers adopt an axial configuration in their functional state, with a preference for the insertion of their GPI-anchored C-termini into virions during their entrapment at the surface of infected cells. Quantitative analysis indicated that an average of ∼80 to 400 tetherin dimers (depending on how many CA molecules are assumed to be present in each virion) associated with each tethered particle. Our findings do not discount the discount the possibility that higher order tetherin multimers, e.g. tetramers, might contribute to tethering, but if such complexes do exist, then they must involve non-covalent interactions between axially configured tetherin molecules and be arranged in such a way that all N-termini and in one membrane (be it virion envelope or cell membrane) and all C-termini are in the opposing membrane.

Previous studies have not resolved the configuration adopted by tetherin during virion entrapment. For example, conflicting results have been obtained in studies where the release of virions was attempted by cleavage of the tetherin GPI anchor using phosphatidyl-inositol-specific phospholipase C (PI-PLC). In one study, the efficiency of virion release induced by PI-PLC treatment was poor (∼20% compared to subtilisin) [22], while other studies indicated that PI-PLC treatment fails to liberate any virions [20], [67]. Second, the failure of reducing agents to release virions would tend to suggest that the equatorial model shown in Figure 1B is incorrect [20]. However, this argument is somewhat confounded by the fact that tetherin molecules are twisted around each other in a dimer, and so breaking the disulphide bonds in an already-formed tether would not necessarily be expected to cause virion release.

One caveat of our assay is that some tetherin dimers might infiltrate particles and yet be uninvolved in restriction. Thus, it is possible that the number of tetherin molecules that we measured to be associated with a virion might be greater than the number of molecules actually involved in virion entrapment. Indeed, previous studies have shown that low levels of complete tetherin molecules can be found in the small number of virions that are released from tetherin-positive cells [20]. However, to be uninvolved in restriction would require that both tetherin N- and C-termini were embedded in virions. If the numbers of tetherin dimers that were inserted into virions in this way was in excess of the numbers of tetherin dimers involved in tethering, with N- and C-termini partitioned separately into virion and cell membranes, then there would be little or no difference in the number of tetherin N- and C-termini found in virions. The fact that we do indeed observe a 3-to 5-fold excess C-termini in tethered virions, argues strongly that most of the tetherin molecules (at least 65–80%) that are tethered-virion associated, have their N- and C-termini separately partitioned into virion and cell membranes. Thus most tetherin molecules must be in the axial configuration with only their C-termini embedded in virions.

Our estimates of the number of tetherin molecules that are associated with tethered virions are several-fold higher than those obtained using super-resolution microscopy approaches (i.e. 4–7 dimers per virion) [22]. At least three factors could account for this discrepancy. First, the microscopy studies use a tetherin-mEosFP fusion protein, that includes a bulky 230 amino acid (∼26 kDa) protein at its N-terminus, appended to the otherwise short (21 amino acid) native tetherin cytoplasmic tail. This could very easily reduce the numbers of tetherin molecules that associate with virions. Second, the estimates made in the microscopy studies correspond to groups of tetherin molecules present at the same location as clusters of Gag molecules that may not represent completely assembled virions. Thus, microscopy studies cannot determine whether the imaged tetherin molecules are in the act of restriction. Conversely, our estimates are based on bona fide tethered virions that are recovered from cells by specific cleavage of the tether. Finally, the cell lines that we used to derived our estimates modestly overexpressed tetherin (1.5- to 3- fold) as compared to HeLa cells, which might have slightly elevated the numbers of tetherin molecules that were associated with virions. In previous studies [22], transfected HeLa cells were used, and the levels of tetherin-mEosFP relative to preexisting endogenous tetherin, or the total (endogenous plus exogenous) levels of tetherin expression were not determined, which could lead to underestimates or overestimates of tetherin association with tethered virions.

Given that virions are trapped not only at the cell surface, but are also linked to each other, it should be expected that both tetherin N- and C-termini would be found in virions. Most likely, the appearance of virions tethered to each other results from the assembly of a virion at a location on the plasma membrane already occupied by a tethered particle. This being so, our finding of a 3- to 5-fold preference for the insertion of C-termini rather than N-termini into virion membranes may represent an underestimate of the true preference. If this is the case, then one might expect that the apparent preference for the insertion of C-termini into virions would become less apparent over time as virion accumulate at the cell surface and the likelihood of a virion assembly at a site already occupied by a tethered virion increased. However, we did not observe such a trend, and thus it remains unclear whether the 3- to 5-fold preference for C-terminus insertion into virions is an accurate number, or an underestimate resulting from virion accumulation.

The biophysical mechanism underpinning the apparent preference for the insertion of GPI-anchored C-termini over TM domain anchored N-termini into virions is unclear at present. Although it is not the predominant scenario, the tetherin N-terminal domain is clearly capable of being incorporated into virions. Indeed, a tetherin molecule lacking the GPI anchor is efficiently incorporated into released virions [19]. Moreover, it is the N- terminus that is targeted by Vpu to block tetherin incorporation into virions [19], [33]. Perhaps the tetherin N-terminal domain acts as a sensor of membrane curvature, driving localization to assembly sites, but the GPI anchor diffuses more freely into virion membranes. Consistent with this idea, recent work indeed indicates that tetherin colocalizes better with HIV-1 Gag proteins that cause membrane curvature than those which do not [68].

There is potential biological utility in preferentially inserting GPI anchored tetherin C-termini rather than N-termini into virions. In such a scenario, the tetherin N-terminus remains available to the cytoplasm of the infected cell, from where it may execute important functions. For instance, virions trapped at the cell surface are internalized and degraded in lysosomes [61], [69]. Moreover, human tetherin appears capable of initiating signaling cascades, particularly when it is engaged in tethering, and in some respects may act as a virion sensor [70]–[72]. Thus, the need to interact with the endocytic machinery and/or initiate signaling might favor a scenario in which tetherin dimers are oriented with their N-termini in the infected cell and their C-termini in the virion membrane.

## Materials and Methods

### Plasmid construction

Tetherin was transiently expressed using pCR3.1 (Invitrogen) based plasmids or stably expressed using pLHCX (Clontech) based retroviral vectors. A human tetherin protein internally tagged with an HA epitope at amino acid 155 and, expressed using pCR3.1 or LHCX vectors, has been described previously [7]. Eight copies of a peptide linker sequence, each comprising the amino acid sequence GGGGS, were inserted immediately C-terminal to the HA tag, to generate the C8 modified tetherin protein (Figure 1D). Similarly, five GGGGS linker units were inserted immediately C-terminal to the tetherin transmembrane domain at amino acid position 50, to generate the N5 modified tetherin protein. Because the BamHI recognition site (GGATCC) encodes a glycine and serine, we incorporated its sequence into the fourth and third linker units for the C8 and N5 proteins respectively. We then used these BamHI sites for the subsequent insertion of a Factor Xa cleavage site (IEGR) to generate the C8Fac and N5Fac proteins (Figure 1D). Thereafter the Flag N5Fac protein was generated by inserting three copies of a FLAG epitope tag at the N-terminus of the N5Fac protein (Figure 1D).

The protein standard used for quantitative western blotting was generated by appending the C-terminus of HIV-1 p24 CA protein with three FLAG epitope tags and an HA epitope tag. Specifically, the p24 CA coding sequence was amplified from the proviral plasmid pNL4-3 using oligonucleotides that encoded the epitope tags, and inserted as an EcoRI-NotI fragment into the multiple-cloning site of pCRV-1, a previously described hybrid expression vector [73] that is derived from pCR3.1 and from a highly modified HIV-1 provirus (V1B). All mutagenesis was accomplished by using overlap-extension PCR.

### Cell culture

Human embryonic kidney (HEK) 293T cells and HeLa-TZM cells expressing CD4/CCR5 and a LacZ reporter gene under control of the HIV-1 LTR were maintained in Dulbecco's Modified Eagle Medium (DMEM) supplemented with 10% FBS and gentamycin (2 µg/ml, Gibco). HEK293T cells were transduced using pLHCX based retroviral vectors expressing genes of interest and selected with hygromycin (50 µg/ml) (MediaTech, Inc) to generate cell lines expressing either the empty vector or epitope-tagged WT or modified tetherin proteins.

### Flow cytometry

The 293T cells stably expressing the modified tetherin proteins and HeLa cells were harvested in PBS plus 5mM EDTA, washed in FACS buffer (PBS plus 2% BSA), and stained with PE anti-human CD317 (tetherin) antibody (Biolegend). Dead cells were excluded by DAPI staining. All data were acquired on an LSR II flow cytometer (Becton Dickinson), and data were analyzed with FlowJo software (Tree Star).

### Virus production

A HIV-1 proviral plasmid that expresses green fluorescent protein (GFP) in place of Nef has been described previously [74]. 293T cells were seeded in 10 cm plates at a concentration of 3×10^6^ cells/plate and were cotransfected the following day using polyethylenimine (PolySciences) with 10 µg of wild-type (HIV-1(WT)) or Vpu-deficient (HIV-1(ΔVpu)) GFP reporter plasmids, along with 1 µg of a VSV-G expression plasmid. The culture medium was replaced the following day. At 48 hours post transfection, the culture supernatants were harvested, clarified by centrifugation at 3000 rpm, and filtered through a 0.2 µm PVDF membrane (Millipore). The viruses were stored at -80°C. Infectious virus titers were determined by inoculating sub-confluent monolayers of 293T cells that were seeded in 96 well plates at 30,000 cells/well with 100 µl of serially diluted supernatants. At 48 hours post infection, the cells were dispersed with trypsin, fixed in 4% paraformaldehyde and analyzed by flow cytometry.

### Virion yield assays

293T cells were seeded in 24-well plates at a concentration of 2×10^5^ cells/well and were cotransfected the following day using polyethylenimine (PolySciences) with 350 ng of wild-type (HIV-1(WT)) or Vpu-deficient (HIV-1(ΔVpu)) proviral plasmids along with varying amounts of a Tetherin expression plasmid (25 ng to 100 ng) and a plasmid expressing YFP (75 ng), to monitor transfection efficiency. In all transfection experiments, the total amount of DNA was held constant by supplementing the transfection with an empty expression vector. The culture medium was replaced the following day. At 48 hours post transfection, the culture supernatants were harvested, clarified by centrifugation at 3000 rpm, and filtered through a 0.2 µm PVDF membrane (Millipore). Infectious virus yield was determined by inoculating sub-confluent monolayers of HeLa-TZM cells that were seeded in 96 well plates at 10,000 cells/well with 100 µl of serially diluted supernatants. At 48 hours post infection, β-galactosidase activity was determined using GalactoStar reagent, in accordance with the manufacturer's instructions (Applied Biosystems). Physical particle yield was determined by layering 700 µl of the virion containing supernatant onto 1 ml of 20% sucrose in PBS followed by centrifugation at 20,000×g for 90 minutes at 4°C. Virion pellets were then analyzed by Western blotting.

### Recovery of tetherin entrapped virions

Cells (HEK293T) stably expressing WT or engineered tetherin proteins were infected with VSV-G-pseudotyped HIV-1(WT) or HIV-1(ΔVpu) GFP at 1 infectious unit per cell in 10 cm dishes. The inoculum was removed 6 h later. At 48 hours post transfection, the culture supernatants were harvested, clarified by centrifugation at 3000 rpm, and filtered through a 0.2 µm PVDF membrane (Millipore). Physical particle yield was determined as outlined above. Simultaneously, the cells were washed with Factor Xa reaction buffer (20 mM Tris·Cl, pH 6.5; 50 mM NaCl; 1 mM CaCl_2_) and incubated with 50 µg of Factor Xa in 5 ml of Factor Xa reaction buffer for 2 hours at 37°C. Alternatively, the cells were washed with with subtilisin A buffer (10 mM Tris ,pH 8.0; 1 mM CaCl_2_; 150 mM NaCl), and treated with 5 ml of 1 µg/ml of subtilisin A (Sigma) for 3 min at room temperature. Subtilisin treatment was stopped using DMEM containing 10% FCS, 5 mM PMSF, and 20 mM EGTA. Thereafter, the supernatants were centrifuged, filtered and virions pelleted as described above, and the cells were lysed for analysis of viral protein expression by Western blotting.

### Peptide-N-glycosidase-F digestion of tetherin

Lysates of cell and liberated virions were denatured with 0.5% SDS at 100°C for 10 minutes and then treated with 1% NP-40 to neutralize the SDS. The lysates were incubated with (or without) 500 U of peptide-N-glycosidase-F (New England Biosciences) at 37°C for 3 hours. Thereafter, the reactions were quenched with SDS-PAGE loading buffer and the samples were analyzed with western blotting.

### Western blot assays

Pelleted virions and cell lysates were resuspended in SDS-PAGE loading buffer, in the presence or absence of β-mercaptoethanol, and resolved on NuPAGE Novex 4–12% Bis-Tris Mini Gels (Invitrogen) in MOPS running buffer. Proteins were blotted onto nitrocellulose membranes (HyBond, GE-Healthcare) in transfer buffer (25 mM Tris, 192 mM glycine). The blots were then blocked with Odyssey blocking buffer and probed with mouse anti-HIV-1 capsid (NIH), rabbit anti-HA (Rockland), and mouse anti-FLAG (Sigma) primary antibodies. For quantitative western blotting, the bound primary antibodies were detected using fluorescently labeled secondary antibodies (IRDye 800CW Goat Anti-Mouse Secondary Antibody, IRDye 680LT Goat Anti-Rabbit Secondary Antibody and IRDye 680LT Goat Anti-Mouse Secondary Antibody; LI-COR Biosciences). Fluorescent signals were detected using a LI-COR Odyssey scanner and quantitated with Odyssey software (LI-COR Biosciences).
